# Efficient high-resolution refinement in cryo-EM with stochastic gradient descent

**Published:** 2024-10-30

**Authors:** Bogdan Toader, Marcus A. Brubaker, Roy R. Lederman

**Affiliations:** 1 MRC Laboratory of Molecular Biology; 2 Yale University; 3 York University

## Abstract

Electron cryomicroscopy (cryo-EM) is an imaging technique widely used in structural biology to determine the three-dimensional structure of biological molecules from noisy two-dimensional projections with unknown orientations. As the typical pipeline involves processing large amounts of data, efficient algorithms are crucial for fast and reliable results. The stochastic gradient descent (SGD) algorithm has been used to improve the speed of ab initio reconstruction, which results in a first, low-resolution estimation of the volume representing the molecule of interest, but has yet to be applied successfully in the high-resolution regime, where expectation-maximization algorithms achieve state-of-the-art results, at a high computational cost. In this article, we investigate the conditioning of the optimization problem and show that the large condition number prevents the successful application of gradient descent-based methods at high resolution. Our results include a theoretical analysis of the condition number of the optimization problem in a simplified setting where the individual projection directions are known, an algorithm based on computing a diagonal preconditioner using Hutchinson’s diagonal estimator, and numerical experiments showing the improvement in the convergence speed when using the estimated preconditioner with SGD. The preconditioned SGD approach can potentially enable a simple and unified approach to ab initio reconstruction and high-resolution refinement with faster convergence speed and higher flexibility, and our results are a promising step in this direction.

## 1 Introduction

We consider the problem of gradient-based optimization for tomographic reconstruction with a particular focus on electron cryomicroscopy (cryo-EM). Stochastic gradient descent (SGD) based optimization methods have become a standard algorithmic framework in many areas due to their speed, robustness, flexibility and ease of implementation, particularly with the availability of fast and robust automatic differentiation libraries. However, in cryo-EM, SGD methods have only really found use in ab initio structure determination, where the goal is only a low-resolution structure. High-resolution structures are then determined by switching to a different form of optimization, typically some form of expectation-maximization (EM), despite the fact that these methods are often slow and require specific modeling assumptions. A natural question which we address here then is why stochastic gradient optimization techniques have not been able to solve the high-resolution optimization problem. Doing so could further speed up data processing for cryo-EM, simplify workflows and unify open research questions. However, perhaps more significantly, using SGD-based optimization methods would allow for more flexibility in modeling the reconstruction problem. Common modeling assumptions (e.g., Gaussian noise, Gaussian priors, rigid structures, discrete Fourier-based structure representations) which may not be optimal but are required by current refinement methods could be relaxed with SGD methods.

In this paper, we show that standard SGD-based methods struggle to accurately determine high-resolution information in cryo-EM due to fundamental properties of the induced optimization problem. We perform a theoretical analysis, in a simplified setting, which shows that the induced optimization problem can suffer from ill-conditioning, which results in arbitrarily slow convergence for standard SGD algorithms. Moreover, our analysis shows that the conditioning may be acceptable at low resolution but becomes worse as resolution increases, explaining why SGD has been successful in the ab initio setting but has yet to be successfully used for high-resolution refinement. While our analysis is in a simplified setting, we argue and empirically verify that this poor conditioning behavior continues to exist in real-world cryo-EM settings. Finally, based on our analysis, we propose a new SGD-based algorithm which, unlike standard methods, exploits higher-order derivatives to improve the conditioning of the problem. Our results demonstrate that this new method is able to overcome the conditioning problem and efficiently and accurately determine high-frequency information.

### 1.1 Background

Cryo-EM enables biologists to analyze the structure of macromolecules in their native state. In comparison with the older method of X-ray crystallography, cryo-EM does not require crystallized samples, allowing one to study larger molecules with complex structure and conformations. Indeed, its potential to uncover the structure and function of macromolecules has been acknowledged by the scientific community: cryo-EM was named the “Method of the year” in 2015 by Nature Methods [1], and its development was the subject of the 2017 Nobel Prize in Chemistry.

The standard cryo-EM single particle analysis (SPA) pipeline involves freezing a biological sample in a thin layer of ice and imaging it with an electron microscope. The imaged sample contains multiple copies of a macromolecule captured in distinct, random and unknown orientations. Following a number of data processing steps, two-dimensional projections of the electrostatic potential of the macromolecule are captured in a stack of images, which we refer to as the particle images. The goal of the cryo-EM SPA pipeline is to reconstruct a three-dimensional volume representing the structure of a molecule from the collected particle images.

In addition to the random orientations, each projection of the volume is shifted off the center of the image by a small, unknown amount, and the particle images are further blurred by a contrast transfer function (CTF) which is image-specific and depends on the settings of the microscope. Moreover, to avoid the damaging of the biological sample by the electron beam, the imaging is done at a low dosage, which results in a poor signal-to-noise ratio (SNR). Therefore, cryo-EM reconstruction requires solving a tomography problem with unknown viewing directions and in-plane shifts, and low SNR. Here we refer to the particle orientations and the in-plane shifts collectively as the pose variables.

Cryo-EM reconstruction approaches implemented in established software packages [2, 3] consist of two separate stages: ab initio reconstruction and high-resolution 3D refinement. Ab initio reconstruction provides an initial, low-resolution estimation of the volume. This is a non-convex problem for which many methods have been developed, but the current state-of-the-art methods are based on stochastic gradient descent algorithms which were first used in the context of cryo-EM in the cryoSPARC software [3, 4]. More recently, a similar approach has been adopted by other software packages such as RELION [5]. After the ab initio step, high-resolution 3D refinement performs further optimization of the volume and generates a high-resolution reconstruction. This usually employs an optimization algorithm such as expectation-maximization [6] to iteratively reconstruct the volume and a search procedure to estimate the pose variables. The EM algorithm has become a standard approach to high-resolution refinement [2, 3, 7, 8] and achieves state of the art reconstructions. However, EM-based methods are computationally expensive, generally requiring full passes through all images at each iteration of refinement and necessitating sophisticated grid and tree search algorithms to reduce the computational costs. Further, they are restricted to several key assumptions including Gaussian noise, a Gaussian prior on structures, a rigid structure, and a discrete Fourier-based representation of the structure.

Recently, a new class of methods for cryo-EM have emerged which aim to reconstruct not just static structures but also conformational heterogeneity, where the reconstructed volume is subject to different kinds of deformations [9–25]. Such methods greatly expand the capabilities of cryo-EM, e.g., with time-resolved cyro-EM [26, 27]. However, existing methods usually require a high resolution structure and known pose variables as input, limiting their applicability. Moreover, they cannot use the standard EM-based optimization approaches, often using neural networks trained using SGD instead, and generally fail to match existing rigid structure refinement approaches in resolution and requiring substantially more computation. While motivated by the promise of capturing conformational heterogeneity, we focus here on the static reconstruction case. Our results suggest that improving the performance of SGD-based methods may be the key to unlocking this new capability of cryo-EM.

### 1.2 Prior work

The Bayesian formulation of the cryo-EM reconstruction problem and its solution via the EM algorithm has a long history, with early works including [7, 28, 29] as well as their implementation for high-resolution 3D refinement in state-of-the-art software such as RELION [2, 8] and cryoSPARC [3]. While early ab initio reconstruction methods involved heuristic approaches such as using a low-pass filtered known reconstruction of a similar structure to the one of interest, mathematically rigorous approaches based on the method of common lines have been developed in [30–32]. The SGD algorithm introduced in a cryo-EM context in [3, 4] showed improved robustness and speed in obtaining ab initio reconstructions from scratch. More recently, the VDAM algorithm, a gradient-based algorithm with adaptive learning rate has been introduced in the latest version of the RELION software [5]. This brief list of cryo-EM reconstruction algorithms is non-exhaustive and we refer the reader to more comprehensive review articles such as [33, 34].

The aforementioned articles view the SGD algorithm and its variants as tools for the ab initio step, while the best results for high-resolution refinement are achieved using the EM algorithm. In this work, we present an analysis of the conditioning of the reconstruction optimization problem and propose a method to improve the convergence of SGD for high-resolution refinement by on-the-fly estimation of a suitable preconditioner. While basic preconditioners are used in cryoSPARC [3] and several strategies for adapting the step size based on second order information are implemented in the VDAM algorithm [5], neither work addresses the conditioning of the problem explicitly, and the preconditioners used are not suitable in the high-resolution regime. In contrast, while we theoretically analyze the reconstruction problem in a simplified setting, our proposed solution is specifically designed to overcome the conditioning issue in a big data, high-resolution setting. We leverage ideas from the machine learning literature [35, 36] to estimate a preconditioner that poses no significant additional computational cost over the ordinary SGD algorithm, does not require a particular initialization or warm start, and is straightforward to incorporate into existing SGD implementations in cryo-EM frameworks. Finally, we provide numerical experiments that show the feasibility of our preconditioning approach for estimating high resolution information.

### 1.3 Outline

The remaining parts of the article are structured as follows. In Section 2, we describe the mathematical setting of the cryo-EM reconstruction problem, as well as current approaches for high-resolution refinement using the EM algorithm and ab initio reconstruction using the SGD algorithm. Section 3 presents the main contributions. In Section 3.1, we analyze the condition number of the linear inverse problem in the simplified setting where the pose variables are known, while in Sections 3.2–3.4 we describe several ideas that are part of a proposed construction of a preconditioner that allows SGD to overcome the conditioning issue. In Section 4, we provide numerical experiments that validate the theoretical contributions, and in Section 5 we conclude with a summary of the main advantages of the proposed approach and motivate a potential extension of this work to the fully general setting.

## 2 Preliminaries

### 2.1 Forward model

The objective of cryo-EM reconstruction is to estimate a three-dimensional volume representing the shape of a molecule v from a stack of particle images ${\left\{x_{i}\right\}}_{i=1}^{N}$. A simple and frequently used model of the image formation process consists of the following steps: each particle image $x_{i}$ is formed by rotating the volume v by a three-dimensional rotation operator $R_{i}$, projecting it along the *z*-axis, applying a small in-plane shift $T_{i}$, convolving the result with a contrast transfer function (CTF) $C_{i}$, and adding Gaussian noise.

This model is often formulated in the Fourier domain to speed up the computation of the projections by taking advantage of the Fourier slice theorem and the fast Fourier transform (FFT). The Fourier slice theorem states that the two-dimensional Fourier transform of a projection of a three-dimensional volume is the intersection of the three-dimensional Fourier transform of the volume with a plane passing through the origin of the coordinate axes, where the projection direction is determined by the orientation of the plane.

Let $v\in C^{M_{v}}$ be the (discretized) three-dimensional volume and $x_{i}\in C^{M_{x}}$, for i=1,...,N, the particle images. Here, $M_{v}$ is the total number of voxels in a M×M×M grid discretization of the volume and, similarly, $M_{x}$ is the total number of pixels in a M×M grid discretization of the particle images^1^. Without loss of generality, we assume that in this vectorized representation of the volume, the first $M_{x}=M\times M$ elements of the volume correspond to the volume slice at z=0 in the Fourier domain. Moreover, note that in this representation, both the CTF $C_{i}$ and the in-plane shift operator $T_{i}$ are diagonal matrices in $C^{M_{x}\times M_{x}}$, since in the Fourier domain, the convolution corresponds to element-wise multiplication and the in-plane shift corresponds to a phase factor. Since the volume and images are defined on discrete grids, the rotated volume and the initial grid coordinates are no longer aligned. Specifically, the value of a volume v acted on by a rotation operator R at the grid coordinates r is given by evaluating the volume v at the rotated coordinates $R^{T}r:(Rv)(r)=v\left(R^{T}r\right)$. However, due to the possible misalignment between the coordinate grid that v is defined on and the rotated coordinates $R^{T}r$, the value of v at $R^{T}r$ must be interpolated using its values at the neighboring grid points.

We define two projection operators that use nearest-neighbor and trilinear interpolation, respectively. In short, projection by nearest-neighbor interpolation assigns to the rotated grid point the value of the volume at the grid point closest to the rotated grid point, while projection by trilinear interpolation performs linear interpolation using the closest eight grid points to the rotated grid point and assigns the resulting value to it, as follows:

#### Definition 2.1 (Projection operator $P_{\phi }$).

*Let* $\phi_{i}\in SO(3)\times C^{2}$ *denote the pose variable encapsulating the rotation matrix* R *and the diagonal shift matrix* T, *and* ${\left\{r_{j}\right\}}_{j=1}^{M_{x}}$ *be the coordinates of the Fourier grid at* z=0. *We define the following projection operators* $P_{\phi }\in C^{M_{x}\times M_{v}}$*:*

1. **Nearest-neighbor interpolation projection operator** $P_{\phi }^{nn}$:
(1) $$\left(P_{\phi }^{nn}v\right)\left(r_{j}\right):=Tv_{k^{*}(j)},$$
  *where* $k^{*}(j)={argmin}_{k\in \left\{1,\ldots ,M_{v}\right\}}{\left\|r_{k}-R^{T}r_{j}\right\|}_{2}$, for all $j=1,\ldots ,M_{x}$. In this case, the matrix $P_{\phi }^{nn}$ has exactly one non-zero element in each row, and therefore ${\left(P_{\phi }^{nn}\right)}^{*}P_{\phi }^{nn}\in R^{M_{v}\times M_{v}}$ *is diagonal.*
2. **Trilinear interpolation projection operator** $P_{\phi }^{\text{tri}}$
(2) $$\left(P_{\phi }^{tri}v\right)\left(r_{j}\right):=Tv_{j}^{*},$$
  *where* $v_{j}^{*}$ is obtained by using trilinear interpolation of v at $R^{T}r_{j}$, for all $j=1,\ldots ,M_{x}$.

As the nearest-neighbor interpolation operator is a diagonal matrix, we will use it to establish theoretical results regarding the conditioning of the reconstruction problem. However, the trilinear interpolation operator is more common in practice, and therefore we will show in numerical experiments that our preconditioner estimation method applies to this case as well.

#### Remark 2.2.

*Since the nearest-neighbor projection operator* $P_{\phi }^{nn}$ *corresponds to a plane slice through the volume passing through the center of the coordinate axes that is approximated by the closest grid points to the plane, it follows from* (1) *that the indices of the non-zero elements in the diagonal matrix* ${\left(P_{\phi }^{nn}\right)}^{*}P_{\phi }^{nn}\in R^{M_{v}\times M_{v}}$ *are the indices of these nearest-neighbor elements in the vectorized representation of the volume* $v\in C^{M_{v}}$. *Moreover, the non-zero (diagonal) elements of* $\left(P_{\phi }^{nn}\right)*P_{\phi }^{nn}$ *are real and positive, as the shift matrix* T *is complex diagonal with elements of unit absolute value*.

#### Remark 2.3.

*An alternative approach to the interpolation given in* Definition 2.1 *is to sample the volume on the rotated grid using non-uniform FFT* [37–39] *as done, for example, in* [40]. *In* [41], *the structure of the matrix involving a projection and a backprojection is leveraged to obtain a fast preconditioner based on a circular convolution* [42].

Given the projection operators defined above, we can state the forward model as:

(3) $$x_{i}=C_{i}P_{\phi_{i}}v+\eta_{i},\;i=1,\ldots ,N,$$

where $P_{\phi_{i}}$ is one of the two projection operators in Definition 2.1, $C_{i}\in C^{M_{x}\times M_{x}}$ is a diagonal matrix corresponding to the CTF, and $\eta_{i}$ is the noise vector in the i-th image, with elements complex normally distributed with variance $\sigma^{2}$. Both the rotation $R_{i}$ and the shift $T_{i}$ are specific to each image $x_{i}$ and not known. The CTF model is the same across all images, with image-specific parameters (e.g., defocus). We assume that the CTF model is known and its parameters have been estimated in advance, and that the noise variance $\sigma^{2}$ is constant across particle images and pixels and has also been estimated in advance.

### 2.2 Bayesian formulation and the EM algorithm

The standard approach to high-resolution 3D refinement in cryo-EM is to solve a Bayesian formulation of the volume reconstruction problem with marginalization over the pose variables. This is solved using the expectation-maximization algorithm [6], which has first been used in the context of cryo-EM for aligning and denoising particle images in [28] and further refined for full volume reconstruction in the software packages RELION [2] and cryoSPARC [3].

Computing the full posterior distribution of the volume v given the particle images ${\left\{x_{i}\right\}}_{i=1}^{N}$ is computationally expensive. Instead, the maximum-a-posteriori (MAP) problem involves computing the volume v that maximizes the (log) posterior:

(4) $$\begin{array}{l} \underset{v\in C^{M_{v}}}{argmax\;}log\;p\left(v|x_{1},\ldots ,x_{N}\right)=\underset{v\in C^{M_{v}}}{argmax}\;log\;p\left(x_{1},\ldots ,x_{N}|v\right)+log\;p(v)\\ \;=\underset{v\in C^{M_{v}}}{argmax}\sum_{i=1}^{N}\;\log\;p\left(x_{i}|v\right)+\log\;p\left(v\right), \end{array}$$

where p(v) is the prior distribution of the volume v and $p\left(x_{i}|v\right)$ is the likelihood probability of observing the image $x_{i}$ given the volume v:

(5) $$p\left(x_{i}|v\right)\propto \int_{SO\left(3\right)\times R^{2}}p\left(x_{i}|\phi_{i},v\right)p\left(\phi_{i}\right)\;d\phi_{i},$$

where $p\left(\phi_{i}\right)$ is the prior distribution of the pose variable $\phi_{i}$ of the particle image $x_{i}$ and $p\left(x_{i}|\phi_{i},v\right)$ is the likelihood of observing image $x_{i}$ given the volume v and pose $\phi_{i}$:

$$p\left(x_{i}|\phi_{i},v\right)\propto \exp\left(-\frac{{\left\|x_{i}\;-\;C_{i}P_{\phi_{i}}v\right\|}_{2}^{2}}{2\sigma^{2}}\right).$$

The volume prior p(v) plays a regularization role in solving the reconstruction problem and, throughout this manuscript, we assume it is Gaussian with variance $\tau^{2}$ for all elements of the volume:

$$p(v)\propto e^{-\frac{\|v{\|}_{2}^{2}}{2\tau^{2}}.}$$

The EM algorithm finds the optimal volume $v^{*}$ in (4) by alternating between two steps at each iteration. The fist step computes the expected value of the posterior of the pose variables given the current estimate of the volume $v^{\left(k\right)}$ (the E step), while the second step computes a new iterate for the volume $v^{\left(k+1\right)}$ that maximizes this quantity (the M step). If the prior is Gaussian, the $v^{\left(k+1\right)}$ can be computed analytically by letting the derivatives be equal to zero. The resulting algorithm performs the following update at iteration k:

(6a) $$\text{E}\;\text{step:}{\;\Gamma }_{i}^{\left(k\right)}\left(\phi \right)=p\left(\phi_{i}=\phi |v^{\left(k\right)},x_{1},\ldots ,x_{N}\right)=\frac{p(x_{i}|\phi ,\;v^{\left(k\right)})p\left(\phi \right)}{\int_{\phi_{l}}\;p\left(x_{i}|\phi_{l},\;v^{\left(k\right)}\right)p\left(\phi_{l}\right)\;d\phi_{l}},$$

(6b) $$\text{M}\;\text{step:}\;v^{\left(k+1\right)}={\left(\sum_{i=1}^{N}\;\int_{\phi }\;\Gamma_{i}^{\left(k\right)}(\phi )P_{\phi }^{*}C_{i}^{2}P_{\phi }\;d\phi +\frac{\sigma^{2}}{\tau^{2}}I_{M_{v}}\right)}^{-1}\left(\sum_{i=1}^{N}\;\int_{\phi }\;\Gamma_{i}^{\left(k\right)}(\phi )P_{\phi }^{*}C_{i}x_{i}\;d\phi \right),$$

where $I_{M_{v}}$ is the $M_{v}\times M_{v}$ identity matrix.

In more general implementations of the EM algorithm in software packages such as RELION and cryoSPARC, the noise variance $\sigma^{2}$ and the prior variance $\tau^{2}$ are not constant across image pixels and volume voxels respectively, and are both estimated at each iteration using the current volume iterate $v^{\left(k\right)}$ and weights $\Gamma_{i}^{\left(k\right)}$.

Since the optimization landscape is non-convex, the EM algorithm can converge to a local maximum different from the MAP estimator [6], so a good initialization is required. Therefore, it is best used for high-resolution refinement, where an initial volume and possibly priors for the pose variables are provided by the ab initio algorithm. However, the E step is particularly expensive at high resolution, as it requires integrating over the entire space of the pose variable $\phi_{i}$ for each particle image in the dataset. In practice, this is performed by employing efficient gridding and search techniques.

### 2.3 Stochastic gradient descent for ab initio reconstruction

The SGD algorithm has become the preferred method for solving the MAP problem (4) in the context of ab initio reconstruction. It was first used in [3] to obtain an initial volume reliably and without requiring a good initialization, as it is the case for EM. The key property that SGD exploits is that the objective function in (4) can be split into separate terms for each particle image, not unlike the loss functions used in the training of deep neural networks. In particular, the objective function (4) can be written as:

(7) $$f\left(v\right)=\frac{1}{N}\sum_{i=1}^{N}f_{i}\left(v\right),$$

where each term $f_{i}$ corresponds to a particle image:

$$f_{i}(v)=log\;p\left(x_{i}|v\right)+\frac{1}{N}log\;p(v)$$

At iteration k, SGD performs the following update:

(8) $$v^{\left(k+1\right)}=v^{\left(k\right)}-\eta_{k}\frac{\;df_{j}(v^{\left(k\right)})}{dv^{\left(k\right)}},$$

where $\eta_{k}$ is the step size at iteration k and the index j of the term (and particle image) used to compute the gradient is chosen uniformly at random.

In practice, a mini-batch of particle images, rather than a single image, is used to compute the gradient at each iteration. The volume update step (8) for the current mini-batch is performed after a refinement step of the pose distribution of each particle image in the current mini-batch given the current volume and data $p\left(\phi_{i}|v^{\left(k\right)},x_{1},\ldots ,x_{N}\right)$, for example by using (6a) similarly to the EM algorithm. Various techniques can be used to speed up the computation time, for example the grid refinement implemented in RELION [2] or the branch-and-bound approach in cryoSPARC [3].

The SGD algorithm has two major advantages over EM. First, it has a lower computational cost per iteration, as it only uses a subset of the dataset, while EM requires a pass through the entire dataset. While computing the integrals in (5) for the images in the mini-batch cannot be avoided, the gridding and searching approaches that EM uses to efficiently sample the space of poses are also beneficial in the implementation of SGD. Second, it has been observed in practice that the noise in the sampled gradient enables SGD to explore the optimization landscape more efficiently before converging to a local minimizer of the objective function, preventing it from becoming stuck in unwanted local minima. Given these advantages, the SGD algorithm is particularly well-suited for ab initio reconstruction, as it has been shown in practice in cryoSPARC [3]. More recently, the gradient-based algorithm VDAM has been introduced for ab initio reconstruction in the RELION software [5]. However, despite the clear benefits in terms of computational cost and convergence speed of stochastic gradient algorithms compared to EM for (low resolution) ab initio reconstruction, EM is still the state-of-the-art approach for high-resolution refinement.

To investigate how the performance of SGD can be improved for high-resolution cryo-EM reconstruction, we first state a convergence result from [43]:

#### Theorem 2.4 (Convergence of the stochastic gradient descent algorithm).

*Suppose that* $f:R^{M}\to R$ *in* (7) *is twice-differentiable and strongly convex and its gradient is Lipschitz-continuous with constant L. Furthermore, we assume that there exists B* ≥ 1 *such that:*

(9) $$\underset{i}{max}\;\left\{\left\|\nabla f_{i}(x)\right\|\right\}\leq B\left\|\nabla f\left(x\right)\right\|.$$

*Let* $v^{*}$ *be the minimizer of* f *and* $v^{\left(k\right)}$ *the iterate generated by the SGD iteration* (8) *with fixed step size* $\eta_{k}=\frac{1}{LB^{2}}$. *Then:*

$$E\left[f\left(v^{\left(k\right)}\right)\right]-f\left(v^{*}\right)\leq {\left(1-\frac{1}{\kappa \left(\nabla^{2}f\left(v^{*}\right)\right)B^{2}}\right)}^{k}\left[f\left(v^{\left(0\right)}\right)-f\left(v^{*}\right)\right],$$

*where* $\kappa \left(\nabla^{2}f\left(v^{*}\right)\right)=\frac{{max}_{j}\;{\;\lambda }_{j}}{{min}_{j}\;{\;\lambda }_{j}}$ *is the condition number of the Hessian matrix* $\nabla^{2}f\left(v^{*}\right)$.

The main implication of Theorem 2.4 is that the rate of convergence of SGD is affected negatively by a large condition number of the Hessian matrix $\kappa \left(\nabla^{2}f\left(v^{*}\right)\right)$. In particular, we can derive that, to reach objective function error $\epsilon =E\left[f\left(v^{\left(k\right)}\right)\right]-f\left(v^{*}\right)$, at least $k^{*}$ iterations are required, where:

(10) $$k^{*}=𝒪(\kappa \left(\nabla^{2}f\left(v^{*}\right)\right)B^{2}log\frac{1}{\epsilon }).$$

Note that we chose this particular result for its simplicity, despite the rather strong condition (9). More general results for the convergence of SGD where this condition is relaxed can be found, for example, in [44–46].

To improve the convergence of SGD, we will precondition it using an approximation of the diagonal of the Hessian matrix, which we will compute using Hutchinson’s diagonal estimator [47]:

(11) $$D=E\left[z⊙Hz\right],$$

where $z\in R^{M_{v}}$ is a vector with elements drawn from a Rademacher(0.5) distribution (the entries of z are 1 or −1 with equal probability) and ⊙ denotes element-wise multiplication.

The central claim of this article is that the condition number of the Hessian of the loss function in the cryo-EM reconstruction problem scales unfavorably with the target resolution of the reconstruction, and therefore it affects the convergence of SGD for high resolution refinement. In the next section, we argue that this is indeed the case and we propose a solution for overcoming this issue in a simplified setting.

## 3 SGD for high-resolution refinement: fixed pose variables

In this section, we study the optimization problem (4) in the setting where the pose variables (the three-dimensional orientations and the in-plane shifts of the particles) are known. While this is a simpler problem that can be solved with other methods, it captures the main difficulty that makes the application of gradient-based algorithms non-trivial at high resolution, namely the large condition number of the Hessian. Therefore, analyzing the reconstruction problem with the known pose variables provides useful insights and directions for approaching the problem in its full generality.

For simplicity and without loss of generality, we assume, like in the previous section, that the variance of the noise $\sigma^{2}$ and the variance of the prior $\tau^{2}$ are fixed and given in advance, and are constant across all images and pixels (in the case of $\tau^{2}$) and across all voxels of the volume (in the case of $\sigma^{2}$). The analysis presented in this section can be generalized to the case where σ and τ are not constant, and the preconditioner estimation we propose has the flexibility to incorporate existing methods for determining these parameters in the reconstruction process. To simplify the notation, we collect these two parameters in one parameter $\lambda =\frac{\sigma^{2}}{\tau^{2}}$, which we will refer to as the regularization parameter.

### 3.1 Condition number

Having access to the true pose variable $\phi_{i}^{*}$ for each image $x_{i}$ is equivalent to taking the prior distribution for the pose variable $\phi_{i}$ in (5) to be a Dirac delta distribution centered at the true value $\phi_{i}^{*}$, namely $p\left(\phi_{i}\right)=\delta_{\phi_{i}^{*}}$. In addition, we assume for simplicity that the images are not deformed by the CTF: $C_{i}=I_{M_{x}}$, for all i=1,...,N. With the regularization parameter λ described above and letting $P_{i}\;:=P_{\phi_{i}^{*}}$, we write the optimization problem (4) as:

(12) $$\underset{v\in C^{M_{v}}}{argmin}\frac{1}{2}\sum_{i=1}^{N}{\left\|x_{i}-P_{i}v\right\|}_{2}^{2}+\frac{\lambda }{2}{\left\|v\right\|}_{2}^{2},$$

Letting f(v) be the objective function in (12) and $f_{i}(v)$ the i-th term:

$$f_{i}\left(v\right)\;:=\frac{1}{2}{\left\|x_{i}-P_{i}v\right\|}_{2}^{2}+\frac{\lambda }{2N}{\left\|v\right\|}_{2}^{2},$$

the optimization problem (12) becomes:

(13) $$\underset{v\in C^{M_{v}}}{argmin}f(v)=\underset{v\in C^{M_{v}}}{argmin}\sum_{i=1}^{N}f_{i}(v),$$

The minimizer of (13) is the point $v^{*}\in C^{M_{v}}$ that satisfies:

(14) $$Hv^{*}=b,$$

where $b=\sum_{i=1}^{N}\;P_{i}^{*}x_{i}$ and $H\in R^{M_{v}\times M_{v}}$ with:

(15) $$H=\nabla^{2}f=\sum_{i=1}^{N}P_{i}^{*}P_{i}+\lambda I_{M_{v}}.$$

Note that, for the problem (12), H is the Hessian of the objective function. The SGD algorithm solves problem (13) by iteratively taking steps in the direction of negative sampled gradient:

$$v^{\left(k+1\right)}=v^{\left(k\right)}-\eta_{k}\nabla f_{j}\left(v^{\left(k\right)}\right),$$

where $\eta_{k}$ is the step size at iteration k and the index j is selected uniformly at random. Its convergence properties are determined by the condition number of the matrix H, as stated in Theorem 2.4.

When the projection matrices $P_{i}$ are the nearest-neighbor interpolation matrices (1) in Definition 2.1, the matrices $P_{i}^{*}P_{i}$ are diagonal with real non-negative elements (see Remark 2.2), thus the Hessian matrix H is also diagonal with real non-negative elements. In this case, its condition number [48] is

(16) $$\kappa \left(H\right)=\frac{{max}_{i}\;H_{ii}}{{min}_{i}\;H_{ii}}.$$

We will now analyze the structure and the condition number of the Hessian matrix H when the projection matrices correspond to nearest-neighbor interpolation, namely $P_{i}=P_{\phi_{i}^{*}}^{nn}$, for i=1,…,N. In order to do so, we introduce two necessary concepts. First, the projection assignment function of a particle image maps each element of the image to the element of the volume whose value is assigned to it by the projection operator.

#### Definition 3.1 (Projection assignment function).

*Let* $P_{i}\;:=P_{\phi_{i}}^{nn}\in C^{M_{x}\times M_{v}}$, i=1,…,N, *be nearest-neighbor interpolation projection matrices given in* Definition 2.1 *and corresponding to the pose variables* $\phi_{i},$ i=1,…,N. *We define the projection assignment function* $\Lambda_{i}:\left\{1,\;2,\ldots ,M_{x}\right\}\to \left\{1,\;2,\ldots ,M_{v}\right\}$ *as the function that maps each pixel index* k *of the* i-*th particle image to the voxel index* $\Lambda_{i}[k]$ *in the volume* v *whose value is assigned by the operator* $P_{i}$ *at index* k. *Namely, we have that*:

$$\left(P_{i}v\right)\left[k\right]=T_{i}\left[k\right]v\left[\Lambda_{i}\left(k\right)\right],\;k=1,\ldots ,M_{x},$$

*where the square brackets notation is used for the value of the image (in the left-hand side) or volume (in the right-hand side) at a particular index* k, *and* $T_{i}[k]$ *is the* k-*th element in the diagonal of the translation matrix* $T_{i}$.

Second, the voxel mapping set of a volume element contains the indices of the images that contain a projection of that volume element.

#### Definition 3.2 (Voxel mapping set).

*For every voxel index* $j\in \left\{1,\ldots ,M_{v}\right\}$, *we define the voxel mapping set* $\Omega_{j}\subseteq \{1,\ldots ,N\}$ *as the set of indices of images that contain a pixel that is mapped by their projection assignment functions* $\Lambda_{i}$ *to* j, *namely:*

$$\Omega_{j}=\left\{i:\exists k\in \left\{1,\ldots ,M_{x}\right\}\;\mathrm{such}\;\mathrm{that}\;\Lambda_{i}(k)=j\right\}.$$

Given the functions $\Lambda_{i}$ and the sets $\Omega_{j}$ defined above, the diagonal elements of the (diagonal) matrix $P_{i}^{*}P_{i}$ are ${\left(P_{i}^{*}P_{i}\right)}_{jj}=0$ if $i\notin \Omega_{j}$ and ${\left(P_{i}^{*}P_{i}\right)}_{jj}\in \{1,\;2\}$ otherwise. We make the following assumptions:

#### Assumptions 3.3.

1. *We assume that each voxel index* j *is mapped at most once by the projection assignment function of an image* $\Lambda_{i}$.
2. *Without loss of generality, we assume that* j=1 *is the index of the voxel corresponding to the center of the coordinate axes. Then, the voxel at* j=1 *is mapped by all projection operators* $P_{i}$, i=1,...,N, *or equivalently*, $\Omega_{1}=N$.

The second assumption above concerns the ordering of the elements in the vectorized representation of the grid, specifically so that the center is mapped to the element at index j=1, while the first assumption simplifies our analysis (at a cost of a factor of at most two in the condition number bound below) by ensuring that the diagonal elements of $P_{i}^{*}P_{i}$ satisfy:

(17) $${\left(P_{i}^{*}P_{i}\right)}_{jj}=\left\{\begin{array}{ll} 0, & \text{if}\;i\notin \Omega_{j},\\ 1, & \text{if}\;i\in \Omega_{j}. \end{array}\right.$$

Then, the full Hessian matrix H is also diagonal, with its diagonal elements given by:

(18) $$H_{jj}=\left|\Omega_{j}\right|+\lambda ,$$

where $\left|\Omega_{j}\right|$ is the cardinality of the set $\Omega_{j}$ and $0\leq \left|\Omega_{j}\right|\leq N$, for all $j=1,\ldots ,M_{v}$.

Finally, Proposition 3.4 below captures the main difficulty of the reconstruction problem, namely the condition number of the Hessian matrix increasing with the resolution.

#### Proposition 3.4 (Condition number bound).

*Let* $M_{x}=M^{2}$ *and* $M_{v}=M^{3}$ *for grid length* M *and let* Assumptions 3.3 *hold for the nearest-neighbor interpolation projection matrices* $P_{i}\;:=P_{\phi_{i}}^{nn}$. *Then, for any fixed number of images* N, *we have that:*

(19a) $$\kappa (H)\geq \frac{N\;+\;\lambda }{N/M\;+\;\lambda },\;\;\;\forall M\leq N,$$

(19b) $$\kappa \left(H\right)=\frac{N\;+\;\lambda }{\lambda },\;\;\;\forall M>N.$$

##### Proof.

Since the matrices $P_{i}$ are the nearest-neighbor interpolation matrices $P_{\phi_{i}}^{nn}$, the matrix H is also diagonal and, according to (16), to compute κ(H) we need to find the largest and smallest elements of H.

Using (18) and Assumptions 3.3, we have that ${max}_{j}\;H_{jj}=H_{11}+\lambda =N+\lambda$.

To compute ${min}_{j}\;H_{jj}$, note that the projection assignment functions $\Lambda_{i}$ for i=1,...,N, map $NM^{2}$ image pixels to $M^{3}$ volume voxels.

For M>N, there are more voxels than total pixels (in all the images), and so there exists a voxel $j^{*}$ such that $\left|\Omega_{j^{*}}\right|=0$. Then, ${min}_{j}\;H_{jj}=H_{j^{*}j^{*}}=\lambda$, and so $\kappa (H)=\frac{N+\lambda }{\lambda }$. For M≤N, there are $NM^{2}$ pixels mapped to $M^{3}$ voxels, and therefore there exists a voxel $j^{*}$ such that $\left|\Omega_{j^{*}j^{*}}\right|\leq NM^{2}/M^{3}=N/M$. Then, ${min}_{j}\;H_{jj}\leq N/M+\lambda$, and so $\kappa (H)\geq \frac{N+\lambda }{N/M+\lambda }$. □

We can also write the bounds in Proposition 3.4 in terms of the radius R in the Fourier domain. If we assume that the number of pixels in a 2D disk of radius R is approximately $\pi R^{2}$ and the number of voxels in a 3D ball of radius R is approximately $\frac{4}{3}\pi R^{3}$ then, following a similar argument, we obtain:

(20a) $$\kappa \left(H\right)≳\frac{N\;+\;\lambda }{\frac{3N}{4R}\;+\;\lambda },\;\;\;\forall R≲\frac{3N}{4},$$

(20b) $$\kappa \left(H\right)=\frac{N\;+\;\lambda }{\lambda },\;\;\;\forall R≳\frac{3N}{4}.$$

More generally, if the ratio of the number of pixels in a projected image and the number of voxels in a volume at a given resolution R is p(R), then the bounds in (19a) and (20a) can be written as:

(21) $$\kappa \left(H\right)\geq \frac{N\;+\;\lambda }{p\left(R\right)N\;+\;\lambda },\;\forall R\;\text{such}\;\text{that}\;\frac{1}{p\left(R\right)}\leq N.$$

While the counting argument above shows that the condition number is large when there are more voxels to reconstruct than pixels in all the particle images, in practice, the condition number grows fast with the resolution due to an additional factor. Specifically, in light of the Fourier slice theorem, each image is used to reconstruct the voxels corresponding to a slice through the volume passing through the center of the coordinate axes. Therefore, the large condition number of the matrix H is also a consequence of the fact that the elements of H corresponding to low-frequency voxels are reconstructed using pixel values in most images, while the elements of H corresponding to high-frequency voxels are “seen” by fewer pixels in the particle images. Each new iteration will provide more information to the low-frequency voxels than to the high-frequency ones (relative to the total number of low-frequency voxels and high-frequency voxels, respectively), which leads to errors being amplified (or corrected) at different rates when solving the inverse problem. Lastly, this problem is exacerbated by preferred orientations of the particles: the orientation angles often do not cover SO(3) evenly in real datasets, causing the Fourier transform of the volume to miss entire slices.

**Figure 1:**
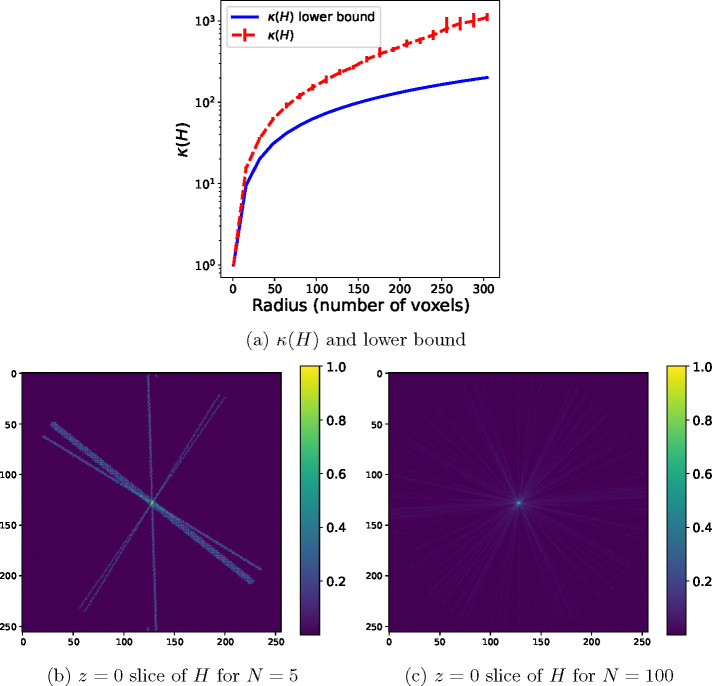
The condition number κ(H) for nearest-neighbor interpolation and no CTF. (a) Lower bound (blue, solid) and average value (red, dashed) of κ(H) for N=10000 images, Fourier radius of R voxels in the range {1,16,32,48,64*,...,*304}, and $\lambda =10^{-8}$. The lower bound is computed using equation (21), where p(R) is computed empirically using spherical and circular shells of radius R. To compute the average value of κ(H) for each R, 10 sets of N uniformly distributed orientations are generated and the average condition number is plotted, as well as error bars showing the minimum and maximum values of the condition number for these sets of orientations. (b,c) The z=0 plane of the diagonal of the nearest-neighbor Hessian matrix H reshaped as a 3D volume, for N=5 and N=100 images, respectively, and grid size 256.

In Figure 1, we illustrate the statement above for the setting of this section, specifically with nearest-neighbor interpolation in the projection operators and no CTF. In panel (a), we show the lower bound on κ(H) given in (21) as a function of the radius in the Fourier space, as well as the condition number for a dataset of N=10000 particle images with uniformly sampled orientations with R ranging from 1 to 304 voxels at intervals of 16 and $\lambda =10^{-8}$. The condition number grows faster than the lower bound due to the effects described in the previous paragraph. To further illustrate the relationship between the number of images and the condition number, we show in panels (b,c) of Figure 1 the Hessian H when using nearest-neighbor interpolation and no CTF when the dataset contains N=5 images (panel (b)) and when the dataset contains N=100 images (panel (c)). This shows how the particle images contribute to a larger fraction of the voxels close to zero than those at a large Fourier radius.

In light of the dependence of the rate of convergence of SGD on the condition number of the Hessian H given in Theorem 2.4 and equation (10), Figure 1(a) suggests that the number of iterations required to reach a certain error grows exponentially with the resolution. Since the root cause is the large condition number at high resolution, we will address this issue by preconditioning the SGD iterations, specifically by using an approximation of the diagonal $D\in R^{M_{v}\times M_{v}}$ of H:

(22) $$v^{\left(k+1\right)}=v^{\left(k\right)}-\eta_{k}D^{-1}\nabla f\left(v^{\left(k\right)}\right).$$

#### Remark 3.5.

*For fixed* σ, τ, *taking* $\lambda =\sigma^{2}/\tau^{2}$ *and known poses, the matrix whose inverse appears in the M step of the EM algorithm in equation* (6b) *is the full Hessian matrix* (15) *of the loss function. Therefore, EM implicitly solves the conditioning issue that is problematic for SGD, and in our preconditioning approach, we aim to approximate, using mini-batches, this gradient scaling that EM applies at every iteration.*

With the facts above regarding the condition number of the Hessian of the loss function, we now proceed to estimate the diagonal preconditioner for this matrix.

### 3.2 Computing the preconditioner

We aim to obtain an approximation of the diagonal of the Hessian matrix H and use it to precondition SGD, which is equivalent to preconditioning the linear system (14) using the Jacobi preconditioner [48]. Motivated by algorithms such as AdaHessian and OASIS in the machine learning literature [36, 49], we estimate the diagonal of the Hessian using Hutchinson’s diagonal estimator [47] stated in (11).

Estimating the diagonal of H using (11) has two practical advantages. First, for any function f, applying (11) only requires computing Hessian-vector products, rather than forming the full Hessian matrix. Indeed, for a function f, a Hessian-vector product is computed efficiently using Jacobian-vector products and automatic differentiation as follows:

(v,z)↦∇(∇f(v))z

Second, the computation can be split into mini-batches so that, at each iteration, only the current mini-batch of images is used for the Hessian-vector product computation.

**Figure 2:**
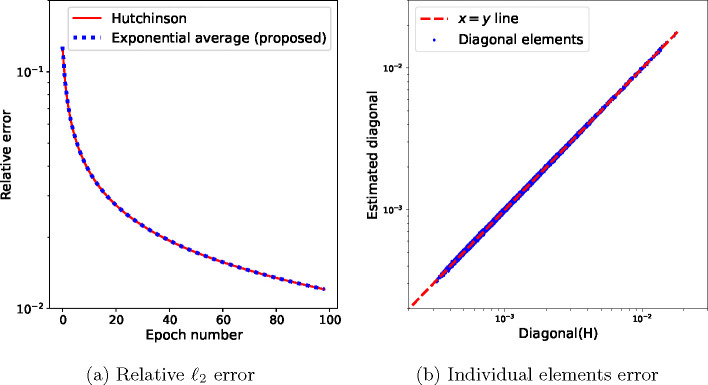
Estimating the diagonal of H using (23), where H is the Hessian matrix of the loss function f in (12) with trilinear interpolation (and therefore, H is not diagonal). (a) Relative $\ell_{2}$ error of the estimated diagonal of H using Hutchinson’s diagonal estimator (11) and the proposed exponential average (23) between an initial estimate $D^{\left(0\right)}$ (here the identity matrix) and the average given by Hutchinson’s estimator. (b) The individual elements of the estimated diagonal plotted against their true values. The accuracy in the smaller elements is lower, as expected for high-frequency elements not mapped by the projection operators of many particle images, i.e. elements at high-frequency indices j with small $\left|\Omega_{j}\right|$.

The update of the preconditioner at the current iteration obtained using the current mini-batch is combined with the estimated preconditioner from the previous iteration using an exponential average as done, for example, in Adam [50], AdaHessian [49] and OASIS [36]. In addition, to take advantage of the fact that the Hessian H in (15) of the objective function (12) is independent of the current iterate when the orientations are known, the exponential average is taken between the estimated preconditioner at the previous iteration and the estimated diagonal using all the samples of Rademacher vectors z drawn up to the current iteration. However, in the more general reconstruction problem with unknown pose variables (4), only the current update would be used. Starting with the identity matrix as the initial estimate, $D^{\left(0\right)}=I_{M_{v}\times M_{v}}$, the update rule for the diagonal estimator $D^{\left(k\right)}$ at iteration k is:

(23a) $$D_{avg}^{\left(k\right)}=\frac{1}{k}\left(z^{\left(k\right)}⊙Hz^{\left(k\right)}\right),$$

(23b) $$D^{\left(k\right)}=\beta D^{\left(k-1\right)}+\left(1-\beta \right)D_{avg}^{\left(k\right)},$$

where β∈(0,1) and the Hessian-vector product is computed using the current mini-batch. An example of the convergence of this estimate over 100 batches of 1000 images each and a total number of *N* = 30000 images is shown in Figure 2.

#### Remark 3.6.

*When the projection operator uses nearest-neighbor interpolation, i.e.* $P_{i}:=P_{\phi_{i}}^{nn}$, *and therefore the Hessian matrix* H *is diagonal, Hutchinson’s estimator with Rademacher samples* (11) *computes the exact diagonal of* H *using a single sample vector z or, if computed using mini-batches, after a single epoch*.

### 3.3 Thresholding of the estimated preconditioner

The combination of variance of the sampled gradient using mini-batches and error in the diagonal approximation of the Hessian, especially in the early iterations, can lead to highly amplified errors in the current iterate $v^{\left(k\right)}$. This is particularly problematic in the case of particles with preferred orientations.

In general, this problem can be avoided by using variance-reduced stochastic gradient methods [51, 52], which require computing full gradients at a subset of the iterations or storing previously computed gradients. However, in a typical cryo-EM setting with large datasets, this has a prohibitive computational cost.

Instead, we propose a simple solution that leverages the particular structure of the cryo-EM reconstruction problem. Given the specific structure of the matrices $P_{i}$ and $P_{i}^{*}P_{i}$ (see Definition 2.1 and equation (17)) and the fact that the projection operators perform slicing in the Fourier domain (according to the Fourier slice theorem), the preconditioned SGD iteration (22) updates the elements of $v^{\left(k\right)}$ corresponding to high-frequency voxels at a lower rate using information in the particle images compared to the low-frequency voxels. Similarly, the elements of the estimated preconditioner $D^{\left(k\right)}$ corresponding to high-frequency voxels in the volume have small values, close to the regularization parameter λ, while the elements corresponding to low-frequency voxels have magnitudes that reflect the large number of images that contribute to the reconstruction of the voxels (see the discussion in the paragraph below equation (21)), and are scaled by the CTF at that particular resolution. In light of (18), this is due to $\left|\Omega_{j}\right|$ being large for low-frequency elements and small for high-frequency elements of the diagonal of H.

This knowledge can be incorporated into the reconstruction algorithm in two ways. One approach is to tune the regularization parameter λ so that it balances the small entries in the preconditioner with the ability to obtain good convergence at high resolution. Alternatively, the same effect can be achieved without interfering with the regularization term by thresholding the smallest elements of the preconditioner, with the benefit of using the structure of the measurement operator in the preconditioner while allowing the freedom to chose the regularization term and parameter by other means, for example as done in RELION [2]. Here, we opt for the latter approach, namely thresholding the elements of the preconditioning matrix at the current iteration $D^{\left(k\right)}$ below a certain value α, chosen as follows.

Let $C_{i}(r)$ be the radially symmetric CTF corresponding to the i-th image, as a function of the Fourier radius r∈[0,R], and let $P_{x}(r)$ and $P_{v}(r)$ be the number of pixels in a 2D Fourier shell at radius r and the number of voxels in a 3D Fourier shell at radius r, respectively. Following the steps in Section 3.1 with non-trivial CTFs and assuming that the particle orientations are uniformly distributed (the details are omitted for brevity), the expected value of an element of the Hessian matrix H at the maximum Fourier radius R is:

(24) $$\overset{‾}{H}=\frac{P_{x}(R)}{P_{v}(R)}\cdot \sum_{i=1}^{N}{\left|C_{i}\left(R\right)\right|}^{2}+\lambda ,$$

Setting the threshold of the smallest elements of $D^{\left(k\right)}$ to $\alpha =\overset{‾}{H}$, the final preconditioner at iteration k is defined as:

(25) $${\overset{ˆ}{D}}_{jj}^{\left(k\right)}=max\{|D_{jj}^{\left(k\right)}|,\alpha \},\;\text{for}\;\text{all}\;j=1,\ldots ,M_{v}.$$

### 3.4 The algorithm

With the preconditioner estimation approach proposed in Section 3.2 and the thresholding described in Section 3.3, we present the full algorithm in Algorithm 1. No warm start is required for the approximation of the diagonal preconditioner, which is initialized with the identity matrix and estimated iteratively. The step size $\eta^{\left(k\right)}$ at each iteration is set using the stochastic Armijo linesearch [35], namely the largest step size η is sought that satisfies the condition:

(26) $$f_{{ℐ}_{k}}\left(v^{\left(k\right)}-\eta D^{-1}\nabla f_{{ℐ}_{k}}\left(v^{\left(k\right)}\right)\right)\leq f_{{ℐ}_{k}}\left(v^{\left(k\right)}\right)-c\cdot \eta {\left\|\nabla f_{{ℐ}_{k}}\left(v^{\left(k\right)}\right)\right\|}_{D^{-1}}^{2},$$

where ${ℐ}_{k}$ is the index set of the current mini-batch, D is the preconditioner, and c∈(0,1) is a hyperparameter. Condition (26) ensures that a sufficient decrease in the objective function is attained over the current mini-batch at every iteration

**Algorithm 1:** 

**Input:** $v^{\left(0\right)},\eta^{\left(0\right)},c$,		
1:	Let $\alpha ={\overset{‾}{H}}_{R}$ as in (24)	
2:	$D_{0}=I_{n}$	
3:	$D_{\mathrm{avg}}=O_{n}$	
4:	**for** k=1,2,... **do**	
5:	Draw index set ${ℐ}_{k}$ and Rademacher(0.5) vector $z^{\left(k\right)}$	
6:	$D_{\mathrm{avg}}=\frac{k-1}{k}D_{\mathrm{avg}}+\frac{1}{k}z^{\left(k\right)}⊙\nabla^{2}f_{{ℐ}_{k}}(v^{\left(k\right)})z^{\left(k\right)}$	▷ Hutchinson’s estimator (23a)
7:	$\;D^{\left(k\right)}=\beta D^{\left(k-1\right)}+(1-\beta )D_{\mathrm{avg}}$	▷ Estimate update (23b)
8:	$\;{\overset{ˆ}{D}}_{jj}^{\left(k\right)}=max\{|D_{jj}^{\left(k\right)}|,\alpha \},\;\;\;\forall j=1,\ldots ,M_{v}$	▷ Preconditioner thresholding (25)
9:	$\eta^{\left(k\right)}=\eta^{\left(k-1\right)}$	
10:	$\;\;v^{\left(k+1\right)}=v^{\left(k\right)}-\eta^{\left(k\right)}{\left({\overset{ˆ}{D}}^{\left(k\right)}\right)}^{-1}\nabla f_{{ℐ}_{k}}(v^{\left(k\right)})$	▷ Stochastic gradient step (22)
11:	**while** $f_{{ℐ}_{k}}(v^{\left(k+1\right)})>f_{{ℐ}_{k}}(v^{\left(k\right)})-c\cdot \eta^{\left(k\right)}{\left\|\nabla f_{{ℐ}_{k}}(v^{\left(k\right)})\right\|}_{{\left({\overset{ˆ}{D}}^{\left(k\right)}\right)}^{-1}}^{2}$ **do**	▷ Line-search (26)
12:	$\eta^{\left(k\right)}=\eta^{\left(k\right)}/2$	
13:	$v^{\left(k+1\right)}=v^{\left(k\right)}-\eta^{\left(k\right)}{\overset{ˆ}{D}}^{-1}\nabla f_{{ℐ}_{k}}(v^{\left(k\right)})$	
14:	**end while**	
15:	**end for**	

## 4 Numerical experiments

In this section, we present numerical experiments^2^ that demonstrate the arguments given in this article regarding the convergence of the SGD algorithm for high resolution cryo-EM reconstruction. In particular, the numerical experiments in this section further verify two claims:

- **Claim 1:** The condition number κ(H) of the Hessian of the loss function is large at high resolution, which leads to slow convergence of the SGD algorithm.
- **Claim 2:** Preconditioning SGD using the diagonal preconditioner as estimated using the tools described in Sections 3.2–3.4 leads to improved convergence at high resolution.

**Figure 3:**
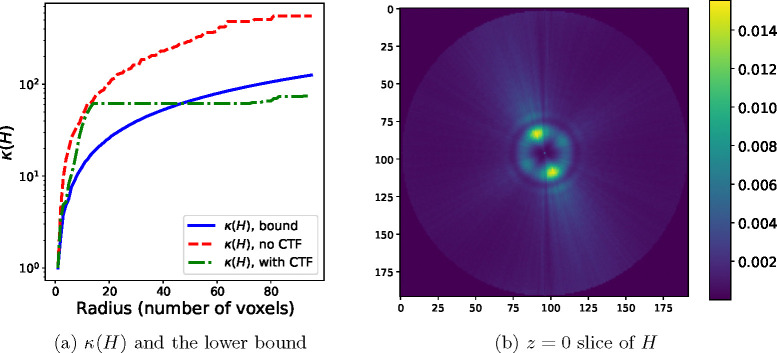
The condition number κ(H) for the experimental dataset used in Section 4, with N=30000 images, grid of dimensions 192 × 192 × 192, and $\lambda =10^{-8}$. (a) Lower bound of κ(H) (blue, solid) computed using (21), the value of κ(H) for the given dataset without CTF (red, dashed) and with CTF (green, dashdot). The condition numbers computed for this plot are for H with nearest-neighbor interpolation. (b) The z=0 plane of the diagonal of the Hessian matrix H for the given dataset, computed using trilinear interpolation and reshaped as a 3D volume. Note the effect of the CTF, in contrast to Figure 1(c), where the particle images are not corrupted by the CTF.

The dataset used in this section is derived from the Electron Microscopy Public Image Archive database entry EMPIAR-10076 of the bacterial ribosome, where N=30000 images of 192 × 192 pixels are selected and whose pose variables have been computed using RELION^3^. The condition number of the reconstruction problem for this dataset with the computed pose variables is shown in Figure 3(a) for increasing values of the maximum Fourier radius R. Nearest-neighbor interpolation has been used for this figure, and therefore the condition number has been computed using (16). The condition number grows quickly with the resolution, and at the maximum radius R corresponding to the grid side length M=192, it is of the order of 10^2^. The bound in Proposition (3.4) and equations (20)–(21), shown as the solid blue line in Figure 3(a), holds for the condition number when no CTF is included, shown as the red, dashed line. Because the CTF has magnitude less than one at the origin and the CTF envelope approaches zero at high spatial frequency, the numerator in (16) is smaller that in the case when no CTF is used, and therefore the condition number computed in this setting, shown in the green dashdot line, is not always larger than the theoretical lower bound. The effect of the CTF can be seen in the cross-section of the diagonal of the Hessian matrix H in Figure 3(b).

To verify the two claims on the dataset described above, we run three algorithms: SGD with no preconditioner, SGD with a preconditioner that has been precomputed in advance using (23), and Algorithm 1, namely SGD with a preconditioner estimated during the refinement process. All algorithms use the stochastic Armijo line-search (26) for the step size adaptation. While the theory in Section 3.1 applies to nearest-neighbor interpolation, where the matrices $P_{i}^{*}P_{i}$ are diagonal (and therefore the optimization problem can be solved easily by other methods), in the numerical experiments presented in this section, the projection operators are implemented using trilinear interpolation, which is often used in practice. In this case, the Hessian matrix H, whose diagonal we estimate and use as a preconditioner, is no longer diagonal.

To evaluate and compare the performance of the three algorithms, we compute the value of the loss function in (12) after each epoch (a full pass through the dataset), as well as the Fourier Shell Correlation (FSC) of each reconstruction with a ground-truth solution, obtained using L-BFGS with 1000 iterations. The FSC, a standard error measure in the cryo-EM literature, is the cross-correlation coefficient between two volumes across three-dimensional shells in the Fourier domain [54]. Specifically, given two volumes u and v in the Fourier space, the FSC at radius r from the origin is defined as

(27) $$FSC\left(r\right)=\frac{\sum_{\ell \in S_{r}}\;u_{\ell }v_{\ell }^{*}}{\sqrt{\left(\sum_{\ell \in S_{r}}\;{\left|u_{\ell }\right|}^{2})(\sum_{\ell \in S_{r}}\;{\left|v_{\ell }\right|}^{2}\right)}},$$

where $S_{r}$ is the set of Fourier voxels in the spherical shell at radius r.

**Figure 4:**
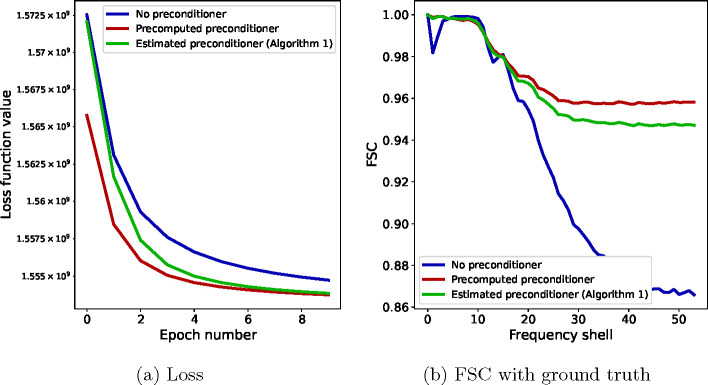
Results of the numerical experiments with trilinear interpolation projection matrices $P_{\phi }^{tri}$ for N=30000 images of 192×192 pixels. Panel (a) shows the loss function value and panel (b) shows the FSC of the final reconstruction with a ground-truth solution for SGD with no preconditioner (blue), SGD with a precomputed preconditioner (red), and Algorithm 1, namely SGD with an estimated preconditioner (green).

Figure 4 shows the results obtained using the three algorithms. In panel (a), we see the convergence of the loss function significantly improved when using the precomputed preconditioner (red) over not using a preconditioner (blue), verifying Claim 1 above. When estimating the preconditioner during refinement using Algorithm 1, the value of the loss (green, solid) is between those of the previous two algorithms and approaches the fully preconditioned SGD as the preconditioner progressively becomes more accurate, verifying Claim 2. In panel (b), we show the FSC between the final reconstructions of each of the three algorithms and the ground truth reconstruction. While at low resolution, the FSC value is high for all algorithms, showing good convergence, at high resolution the FSC degrades for SGD with no preconditioning (blue), while the two preconditioned algorithms have a higher value, closer to one.

**Figure 5:**
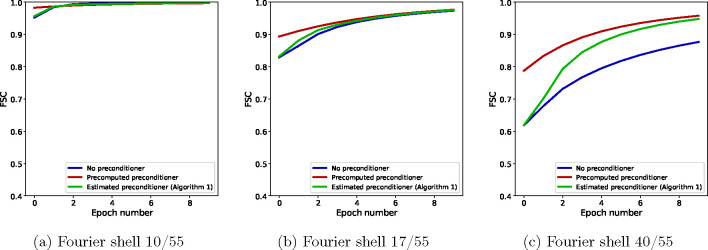
FSC for individual Fourier shells across epochs for a low-frequency shell (a), a middle-frequency shell (b) and a high-frequency shell (c). While preconditioning is not required at low resolution, as seen in (a), its effect becomes increasingly apparent at higher resolution. Note that, since in these experiments the projection matrices use trilinear interpolation, the Hessian matrix H is not diagonal, and yet the diagonal preconditioner is effective.

**Figure 6:**
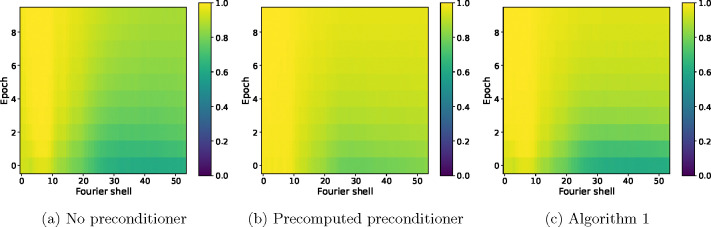
FSC for individual Fourier shells as a function of the Fourier shell index and epoch number for SGD with no preconditioner (a), SGD with a precomputed preconditioner (b) and SGD with an estimated preconditioner (c), where the projection operation is implemented using trilinear interpolation.

For further insight into the difference between SGD without a preconditioner and SGD with a precomputed preconditioner at low and high resolution, as well as how the estimated preconditioner behaves in relation to them, we show in Figure 5 the FSC with the ground truth for the three algorithms at specific Fourier shells and across epochs. Panel (a) shows the FSC for a low-frequency Fourier shell, panel (b) shows a medium-frequency shell, and panel (c) shows a high-frequency shell. At low resolution, all three algorithms are almost indistinguishable, while at medium resolution, the non-preconditioned SGD shows slower convergence than the preconditioned SGD, with SGD with the the estimated preconditioner quickly approaching the accuracy of the fully preconditioned SGD. The advantage of using a preconditioner is seen most clearly at high resolution, where the FSC of non-preconditioned SGD is much lower than the other two algorithms. SGD with an estimated preconditioner converges more slowly than at medium resolution, but eventually approaches the FSC of SGD with a precomputed preconditioner. The small difference in the FSC at the last epoch between the two preconditioned algorithms is likely due to a combination of factors, for example the limited accuracy of the preconditioner estimate after only 10 epochs. Lastly, we show in Figure 6 a complete overview of the shell-wise FSC for each algorithm using a heat map of the correlation as a function of the Fourier shell number and the epoch number.

**Figure 7:**
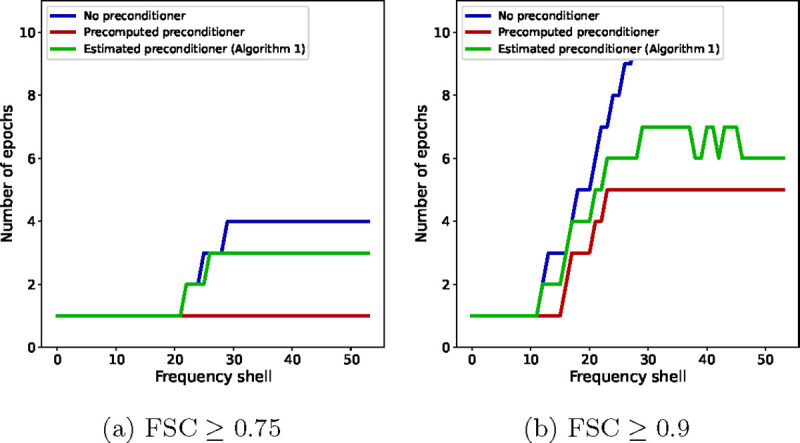
The number of epochs after which the FSC with the ground truth, for individual frequency shells, is greater than θ for SGD with no preconditioner (blue), SGD with a precomputed preconditioner (red) and SGD with an estimated preconditioner (green). (a) θ=0.75 and (b) θ=0.9.

Finally, in light of equation (10), the number of iterations or epochs required for a gradient-based algorithm to achieve an accuracy ϵ scales linearly with the condition number of the Hessian matrix H. We show in Figure 7 the impact of the condition number on the number of epochs required by each of the three algorithms to reach a certain FSC value in our experiments, for each Fourier shell. As the condition number of the preconditioned algorithms scales better with the resolution, the plots show the number of iterations growing more slowly with the resolution for the preconditioned algorithms than in the non-preconditioned case.

## 5 Outlook and conclusion

In this article, we analyzed the the conditioning of the cryo-EM reconstruction problem with a view towards applying stochastic gradient methods efficiently at high resolution. The proposed preconditioner construction and the numerical experiments performed show promising results for high-resolution 3D refinement. While the analysis and experiments presented hold in the special case when the pose variables are known, this simplified setting captures the main difficulty of applying gradient-based algorithms at high resolution, namely the large condition number of the Hessian of the loss function due to the particular structure of the projection operator in the Fourier domain.

This proof-of-concept work shows the potential of the SGD algorithm for the more general cryo-EM reconstruction problem. There are a number of benefits that such an approach would provide:

1. The main advantage is the improved convergence speed. While the EM algorithm requires a full pass through the entire dataset at each iteration, SGD methods only use a mini-batch of the particle images. By estimating a preconditioner during the reconstruction process, the convergence of SGD improves at high resolution, while also benefiting from the speed of using mini-batches. In contrast, EM implicitly computes the same preconditioner, but at an increased computational cost due to requiring the entire dataset at each iteration.
2. A critical component of the current EM approaches is the $\ell_{2}$ regularizer, which makes the maximization step computationally tractable. SGD, on the other hand, is compatible with other regularization methods, and one could take advantage, for example, of the Wilson prior [55] and learned regularization methods [56–58].
3. While the numerical experiments presented here illustrate the performance of the estimated preconditioner with a simple SGD implementation, the preconditioner is compatible with existing and more sophisticated stochastic gradient methods used in established cryo-EM software. Unifying the steps of the ab initio reconstruction and high-resolution refinement using a single algorithm is not only more consistent conceptually, but also a practical improvement, allowing a more streamlined implementation.

We defer to future work the analysis and the preconditioner construction in the general case, where the pose variables are not known. The main difficulty in the general case over the setting of our analysis is that, when marginalizing over the unknown poses (see (5)), the objective function is no longer quadratic. Therefore, the Hessian depends on the current volume iterate. However, it is expected that the pose prior distributions are already narrow at high resolution and do not vary considerably from one epoch to another. Moreover, at each iteration, only a subset of the pose variables are updated (those corresponding to the particle images in the current mini-batch), and existing efficient pose sampling techniques can be used. Therefore, an approach to estimate the preconditioner in the general case based on similar ideas to the ones presented in this article is a promising future direction.

## Footnotes

1 Throughout this article, we will use the words “voxel” and “pixel” to refer to the elements of a discretized volume and image respectively, in the Fourier domain.

2 The code for reproducing the numerical experiments is available at https://github.com/bogdantoader/simplecryoem.

3 The parameters were first obtained from the CryoDRGN tutorial [11], converted to RELION format using Pyem [53] and then further processed in RELION [2].
